# Neural Dynamics of Shooting Decisions and the Switch from Freeze to Fight

**DOI:** 10.1038/s41598-019-40917-8

**Published:** 2019-03-12

**Authors:** Mahur M. Hashemi, Thomas E. Gladwin, Naomi M. de Valk, Wei Zhang, Reinoud Kaldewaij, Vanessa van Ast, Saskia B. J. Koch, Floris Klumpers, Karin Roelofs

**Affiliations:** 1 Donders Institute for Brain Cognition and Behaviour, Centre for Cognitive Neuroimaging, Radboud University, Nijmegen, 6525EN Netherlands; 2 Behavioural Science Institute, Radboud University, Nijmegen, 6526HR Netherlands; 3 Department of Psychology and Counselling, University of Chichester, Chichester, West Sussex P019 6PE United Kingdom; 4 Department of Clinical Psychology, University of Amsterdam, Amsterdam, 1018WT Netherlands

## Abstract

Real-life shooting decisions typically occur under acute threat and require fast switching between vigilant situational assessment and immediate fight-or-flight actions. Recent studies suggested that freezing facilitates action preparation and decision-making but the neurocognitive mechanisms remain unclear. We applied functional magnetic resonance imaging, posturographic and autonomic measurements while participants performed a shooting task under threat of shock. Two independent studies, in unselected civilians (N = 22) and police recruits (N = 54), revealed that preparation for shooting decisions under threat is associated with postural freezing, bradycardia, midbrain activity (including the periaqueductal gray-PAG) and PAG-amygdala connectivity. Crucially, stronger activity in the midbrain/PAG during this preparatory stage of freezing predicted faster subsequent accurate shooting. Finally, the switch from preparation to active shooting was associated with tachycardia, perigenual anterior cingulate cortex (pgACC) activity and pgACC-amygdala connectivity. These findings suggest that threat-anticipatory midbrain activity centred around the PAG supports decision-making by facilitating action preparation and highlight the role of the pgACC when switching from preparation to action. These results translate animal models of the neural switch from freeze-to-action. In addition, they reveal a core neural circuit for shooting performance under threat and provide empirical evidence for the role of defensive reactions such as freezing in subsequent action decision-making.

## Introduction

Life-or-death shooting decisions by police officers and other armed professionals demand fast switching between distinct defensive behaviours, such as freezing –known to facilitate vigilant assessment of the situation– and active fight-or-flight responses. Although these professionals are generally well trained to cope with threat, shooting accuracy has been reported to decrease from over 90% during training to 50% during real-life threat^1,2^. These stress-induced reductions in performance illustrate the need to understand the neurophysiological mechanisms underlying such critical shooting decisions in order to optimize performance under high threat.

Throughout evolution, predator threat has shaped distinct defensive behavioural patterns that are dynamically supported by the autonomic nervous systems (ANS)^3–5^. The detection of threat activates species-specific defensive reactions, such as freezing behaviour, which is characterized by the cessation of bodily movement^6^. Freezing behaviour is associated with both parasympathetic and sympathetic ANS activity. In human studies, movement cessation as a result of threat processing is typically associated with concomitant heart rate decelerations or bradycardia^7^, although this association appears to be species– as well as context–dependent^8,9^. Interestingly, bradycardia has been linked to fast perceptual processing of threat-related information in humans^10,11^ and has been suggested to support decision-making on the most optimal defensive action^12^.

In rodents, neurobiological evidence highlights the role of the midbrain periaqueductal gray (PAG) and the central nucleus of the amygdala (CE) in freezing reactions. The PAG receives downstream connections from the CE that signal the detection of threat and initiate freezing reactions^13^. For example, it has been shown that stimulation of both the CE and the ventral PAG initiates behavioural freezing as well as bradycardia, whereas lesioning of the same areas eliminates these preparatory responses^14,15^. In humans, threat-induced bradycardia is also associated with both increased PAG activity and PAG-amygdala connectivity^16^, but not always with amygdala activity^17^. In spite of its demonstrated importance in human vigilance as well as the theoretical notions that it may be a crucial preparatory stage for the switch to action^18^, defensive reactions such as freezing are often described as passive threat reactions^19^. They have mostly been studied in passive paradigms where active defence is not an option. As a consequence, the role of freezing and the PAG in action preparation remains largely unknown.

When threat becomes more imminent and the possibility of escape or attack exists, a switch to active defence such as fight-or-flight is required, necessitating parasympathetic withdrawal and sympathetic dominance^20,21^. Candidate brain regions associated with the switch to defensive actions and associated heart rate acceleration or tachycardia are the amygdala and the medial prefrontal cortex (mPFC)^22,23^. Interestingly, stimulation studies in rodents indicate that specific neurons in the CE switch behavioural responses to a threatening stimulus from freezing to overt defensive actions^24–26^. Importantly, the mPFC is activated during this switch^24^ and mPFC lesions have been shown to block the switch from freezing to avoidance behaviours^26^. However, whether humans use similar networks to switch from reactive to active defensive behaviours is unclear.

Based on this previous work, our first hypothesis was that PAG activity -associated with bradycardia under threat-potentatied anticipatory freezing- relates to action preparation. Second, we hypothesized that the amygdala and mPFC facilitate a switch from these preparatory reactions to subsequent actions with concomitant tachycardia.

**Figure 1:**
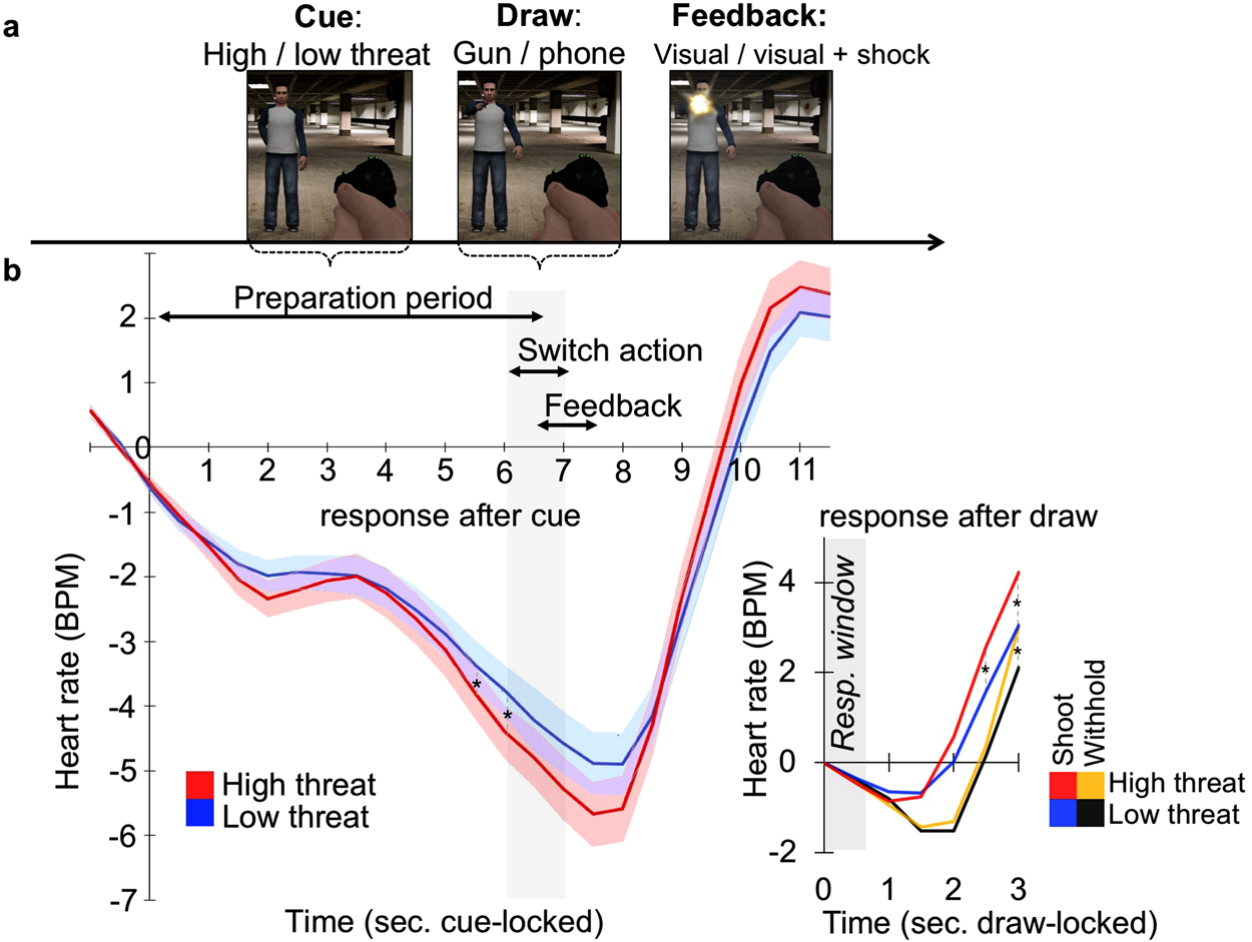
(**a**) Trial sequence of the shooting task: One of two distinct human opponents is presented signalling threat of shock (high threat cue) or shock safety (low threat cue). After a jittered preparation period, the opponent either draws a gun (requiring a shooting response by button press) or a mobile phone (requiring the withholding of a response). Too slow, too fast or incorrect shooting decisions are followed by visual feedback and an additional electric shock after the high threat cue, but not after the low threat cue. (**b**) Average cardiac response across participants during the full trial time-locked to the cue onset (left) and specifically locked to the draw onset (right). Preparation periods were jittered in duration, thus threat effects on preparation and action periods were tested separately on cue and draw-locked data respectively. Heart rate results in beats-per-minute (BPM) indicate a preparatory state of heart rate deceleration followed by a switch to heart rate acceleration in the seconds following the gun draw when critical action was required. Threat of shock potentiated these dynamics (red vs. blue line; asterisks indicating pair-wise significance between high and low threat conditions (Wilcoxon signed-rank test) p < 0.05). Shaded areas represent standard errors of the mean.

During a shooting task, we combined autonomic physiological measurements with functional magnetic resonance imaging (fMRI -see Fig. 1a) and with additional posturographic assessments on a force platform outside the scanner (see SI Appendix). The shooting task was specifically designed to study how shooting decisions evolve from initial preparation to final action. Participants had to shoot or withhold responding, depending on whether an opponent was armed (drawing a gun) or unarmed (drawing a phone). Real-life threat was mimicked by the threat of electric shock that could be avoided by a correct and timely response. We tested our hypotheses in two independent studies encompassing an unselected group of civilians (Study 1 – pre-study N = 22) and police recruits (Study 2; N = 54), respectively.

## Results

Below results are reported for both studies, which is done to illustrate the robustness of effects given reproducibility concerns in science^27,28^. However, given that effects were highly similar and not expected to be different in both samples, we provide no direct comparisons between studies. Figures are only included in the main text for the larger study in police recruits (Study 2) for conciseness. Figures for the smaller Study 1 are reported in the SI Appendix.

### Threat magnitude amplifies freezing-related bradycardia during preparation and action-related tachycardia during shooting

Average heart rate across participants during the task was 68.5 beats-per minutes, in line with alert wakefulness in supine position. We analysed beat-to-beat changes in response to high and low threat cues to verify that threat induces bradycardia during preparation for decisions under threat, and tachycardia during the switch to action (Fig. 1).

#### Preparation

The preparation for shooting decisions -as signaled by the cue onset- slowed down heart rate responses (time main effect [relative to cue onset] Study 1: F_(1.28,21.82)_ = 10.36 p = 0.002; Study 2: F_(1.6,65.69)_ = 55.43 p < 0.001). This result is in line with studies showing that independent of overt threat, selective attention and response preparation produce bradycardia^29,30^. However, as expected, high threat (vs. low threat) deepened this heart rate deceleration significantly (Fig. 1). While this pattern was observed in both studies, the corresponding interaction did not reach significance in Study 1 (F_(1.47,24.98)_ = 2.19 p = 0.14). Post-hoc power calculations however indicated that Study 1 with its’ smaller sample size had lower than recommended power to detect this effect (N = 18, power (1-β = 0.59) for alpha = 0.05). Indeed, threat-related bradycardia in anticipation of shooting was confirmed in the larger Study 2 (cue [high vs. low threat]x time: F_(1.91,78.45)_ = 3.87 p < 0.027, Fig. 1), with post-hoc tests indicating a larger heart rate deceleration in the high threat condition at timepoints 5.5 s and 6.0 s (Wilcoxon signed-rank test for non-normal distributions p = 0.036 and p = 0.01, respectively). These data replicate previous findings in humans showing that anticipation of threat triggers robust fear bradycardia^16,23,31^. Corroborating this, and in line with previous work on human freezing^29^, data from the participants in Study 2 who performed the same shooting task on a stabilometric platform demonstrated significant movement cessation during preparation for shooting, which was strongest during high threat (see SI Appendix, Supplementary Fig. 2). This threat-induced body-sway reduction was significantly correlated with threat-potentiated heart rate deceleration (Rs = 0.37, p = 0.006, Supplementary Fig. 2).

#### Switch to action

The switch from preparation to shooting –as initiated by the draw of the opponent– was in turn associated with accelerated heart rate (time main effect [relative to draw onset] Study 1 F_(5,60)_ = 12.16 p < 0.001; Study 2 F_(5,195)_ = 95.87 p < 0.001), and also this effect was stronger under threat of shock compared to shock safety (cue [high vs. low threat] x time: Study 1: F_(2.38,28.52)_ = 4.6 p < 0.014; Study 2: F_(2.23,86.93)_ = 11.16 p < 0.001, Fig. 1). Follow-up post-hoc tests (shooting: high vs. low threat) indicated threat potentiation of tachycardia at timepoints 2.0 s (only in Study 1 t_(12)_ = −2.72 p = 0.02; Study 2 t_(39)_ = −0.25 p = 0.8), 2.5 s (Study 1 t_(12)_ = −2.38 p = 0.04; Study 2 Wilcoxon: p = 0.003) and 3.0 s (Study 1 t_(12)_ = −2.11 p = 0.056; Study 2 Wilcoxon p = 0.001). As expected, heart rate also accelerated more for shooting than for withhold responses (draw [shoot vs. withhold] x time: Study 1: F_(1.91,22.89)_ = 9.48 p = 0.001; Study 2: F_(1.72,67.18)_ = 16.16 p < 0.001) and this effect was confirmed by post-hoc tests at timepoints 1.5 s (Study 2 t_(39)_ = 3.19 p = 0.003), 2.0 s (Study 1 t_(12)_ = 2.7 p = 0.02; Study 2 Wilcoxon p < 0.001), 2.5 s (Study 1 t_(12)_ = 3.0 p = 0.01; Study 2 Wilcoxon p < 0.001) and 3.0 s (Study 1 t_(12)_ = 2.61 p = 0.02; Study 2 Wilcoxon p = 0.003, see SI Appendix, Supplementary Fig. S1 for all results). We could rule out that enhanced heart rate acceleration in the high threat condition would merely be a result of the enhanced deceleration by repeating the analyses with a baseline centred on the cue (instead of the draw, see Supporting Information).

Thus, together heart rate results demonstrated that shooting decisions involved a switch from preparatory bradycardia to action-related tachycardia, which is magnified under threat of shock and present both in police recruits and untrained control subjects.

### Threat of shock increases tendency to shoot

**Figure 2:**
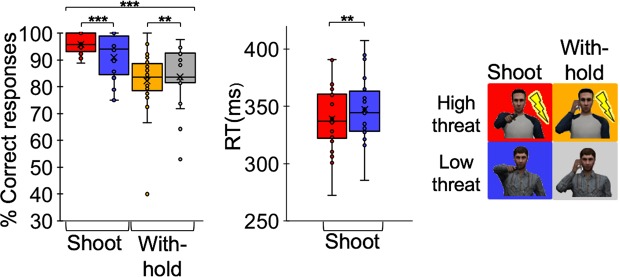
On average, high threat facilitated faster and more accurate responding in shooting trials (when the opponent drew the gun) but increased errors in withhold trials (when the opponent drew the phone) across participants. Non-parametric Wilcoxon signed-rank tests were performed to minimize any potential non-normality and outlier concerns. Asterisks indicate pair-wise significance (Wilcoxon signed-rank test for accuracy and RT) **p < 0.01, ***p < 0.001.

Analyses of behavioural results (accuracy and reaction time [RT]) indicated that threat of shock was associated with trigger-prone behaviour. Depending on whether the task required shooting or withholding, opposing threat effects on accuracy were observed (cue [high vs. low threat] x draw [shoot vs. withhold] Study 1 F_(1,21)_ = 5.53 p = 0.029; Study 2 F_(1,53)_ = 23.72 p < 0.001, Fig. 2). When required to shoot, threat of shock (vs. shock safety) increased shooting accuracy (Wilcoxon: Study 1 p = 0.04; Study 2 p < 0.001). In contrast, when required to withhold from shooting, decreased shooting accuracy during threat of shock (vs. shock safety) was observed (not in Study 1: T_(21)_ = −0.67, p = 0.51; but in Study 2: Wilcoxon p = 0.008). Correct shooting responses were faster during threat of shock (vs. shock safety) (Study 1: T_(21)_ = −4.47, p < 0.001; Study 2: Wilcoxon p = 0.001).

In sum, threat of shock potentiates trigger-prone behaviour by facilitating faster, accurate responding when shooting is required. However, this comes at the cost of increased errors when it is necessary to withhold shooting (see SI Appendix, Supplementary Fig. 3).

### Threat-anticipatory midbrain PAG-centered activity is associated with action preparation under high threat

In the BOLD functional MRI data, we first examined which brain regions were activated during preparation for action under threat of shock (versus shock safety) using whole brain analysis. Significant activations were mostly found in regions associated with threat appraisal and action preparation, including the midbrain (thalamus, hypothalamus and a region consistent with the PAG), striatum (including the putative bed nucleus of the stria terminalis), supplementary motor cortex (SMA), dorsal ACC, and temporal as well as occipital regions (see SI Appendix, Supplementary Table 1). Activations overlapped extensively between Study 1 and Study 2 (see SI Appendix, Supplementary Fig. 4), indicating the robustness of this core neural circuit and the reliability of our measurements.

**Figure 3:**
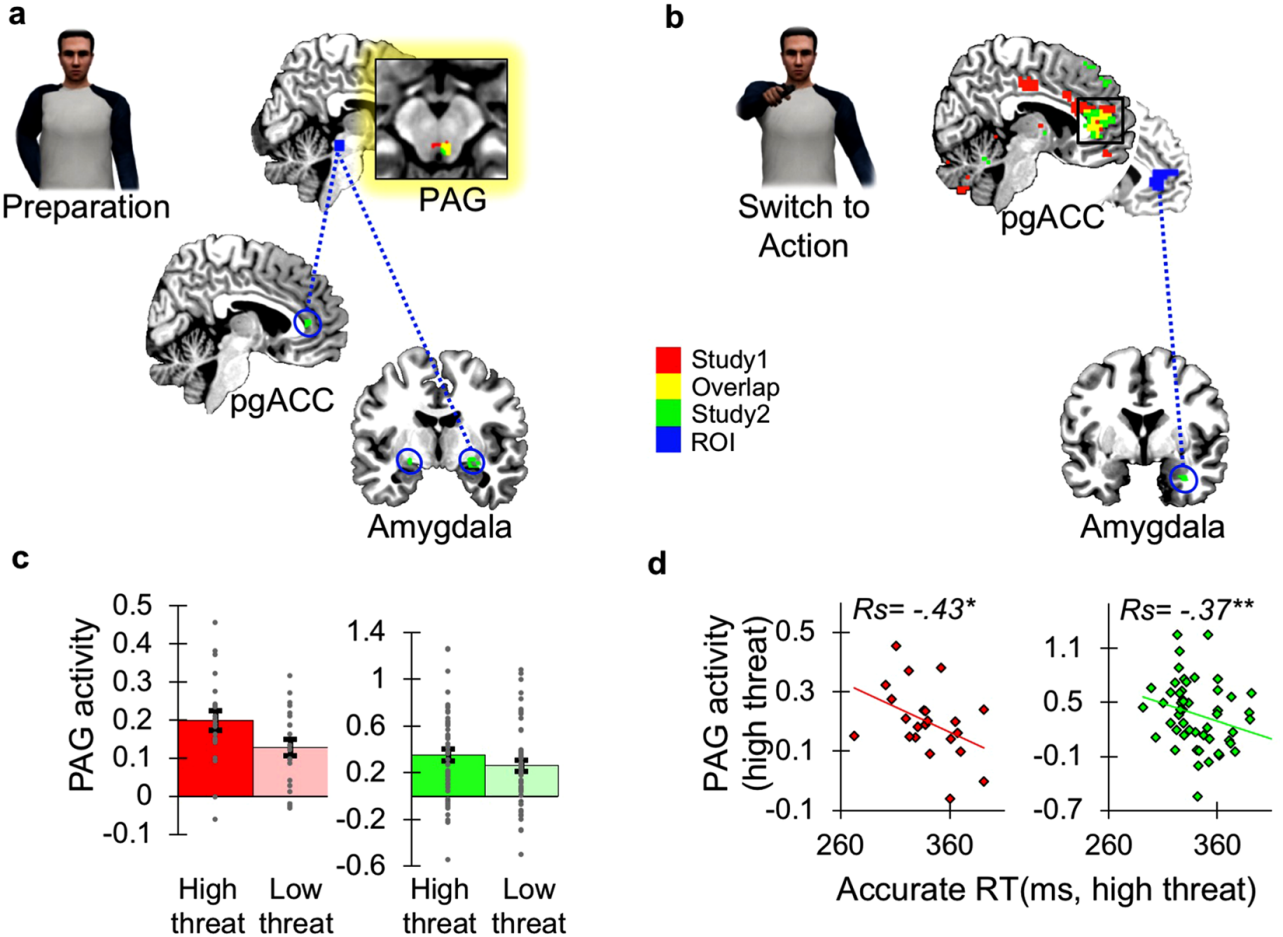
(**a**) Preparation for action under high threat elicited activity in the periaqueductal gray (PAG) compared to low threat trials in both studies, as well as connectivity between the PAG (seed region) and perigenual anterior cingulate cortex (pgACC) and amygdala. (**b**) There was stronger pgACC activity during the switch to action in high threat compared to low threat trials in both studies, as well as stronger pgACC amygdala connectivity under high threat. PAG and amygdala activation were also found, but irrespective of threat magnitude. (**c**) Anatomically derived average PAG parameter estimates during preparation under high threat and low threat in Study 1 (red, N = 22) and Study 2 (green, N = 54) (**d**) Stronger PAG activity during preparation under high threat was related to faster shooting on correct trials in Study 1 (red) and Study 2 (green), analysed with two-sided Spearman Rho tests. All brain results are p < 0.05 FWE corrected for multiple comparisons. For illustrative purposes, all results are visualized with uncorrected threshold of p < 0.01, except for the activation overlap in the small PAG region where an uncorrected threshold of p < 0.05 is used. *p < 0.05, **p < 0.01.

To more specifically test our first hypothesis that human PAG activity –previously linked to freezing– is associated with the preparation for action, we additionaly performed a more anatomically focused analysis. Using an apriori anatomical definition of the PAG, we found stronger PAG activation during preparation under threat of shock relative to shock safety (small volume corrected (SVC) for the PAG: Study 1 [4 −26 −11] p = 0.022; Study 2 [4 −26 −17] p = 0.025, [−2 −30 −17] p = 0.048, see Fig. 3a,c for activation overlap in both studies). Following previous human work on the relation between neural responses and bradycardia during freezing reactions, we checked whether stronger PAG BOLD increases were associated with stronger heart rate decelerations during preparation under threat of shock, which was indeed the case in the police sample (Study 2 high threat: Rs_(42)_ = −0.31 p = 0.044), but not in the smaller Study 1 (Rs_(18)_ = 0.14 p = 0.56 see SI Appendix, Supplementary Fig. 5). A post-hoc power calculation suggested a lack of power in Study 1 based on the effect size given by Study 2 (power: 1 − $\beta$ = 0.29, N = 22, alpha = 0.05). More importantly, in line with a role for freezing in action preparation, stronger PAG BOLD increases during preparation consistently correlated with faster shooting during threat of shock on correct trials (Study 1: Rs = −0.43 p = 0.048; Study 2: Rs = −0.37 p = 0.005 -see Fig. 3d), but not with erroneous shooting decisions including false alarms on withhold trials or misses on shoot trials (Study 1: Rs = 0.1 p = 0.67, Study 2: Rs = 0.09 p = 0.52). These findings match with the hypothesized role of the PAG in freezing and peak activations are largely in line with previous findings^16,32^, although it deserves to be noted that influences from other midbrain nuclei cannot be fully ruled out here.

Together, these results suggest that threat-anticipatory midbrain activity, the source of which appears PAG centered, is associated with increased action preparation under high threat. Interestingly, and similar to this midbrain activity, anticipatory body sway reductions that were measured in the same participants using the same shooting task on a stabilometric platform outside the scanner in Study 2, were also related to threat-related reductions in reaction times under high threat (Study 2 cue [high vs. low threat]: Rs = 0.32, p = 0.02). This result further supports the role of freezing in action preparation.

### Perigenual ACC as the neural switch from preparation to action under high threat

For the second aim of the study, we identified brain regions in which activity was associated with the switch from defensive preparation to action under high threat conditions. In both studies, whole brain analyses indicated that activation of the perigenual anterior cingulate cortex (pgACC) was in line with such a role; it was the only brain region that was specifically more active at the moment that participants were required to shoot during threat of shock (as opposed to shock safety), and this effect was present in both studies (FWE whole brain corrected: Study 1 [−12, 43,1] p < 0.001; Study 2 [11, 46, 13] p = 0.002, see Fig. 3c). The switch-related pgACC activation was not correlated with the observed tachycardia or behavioural responses, presumably because these were influenced by the preparatory responses directly preceding this activation. Although we did not observe BOLD increases in the amygdala within this contrast, the amygdala appeared to be involved in the switch to action irrespective of threat of shock. Comparison of shooting and withhold responses in the larger Study 2 indeed showed significant activation of the left amygdala (SVC [28, −6, −14] p = 0.005). Consistent with recent findings in rodents showing that local circuits in the PAG may also mediate the switch to defensive actions^33^, the PAG was activated on shooting trials as opposed to withhold responses irrespective of threat of shock (Study 1: SVC [4 −30 −8] [−2 −33 −80] both p < 0.001; Study 2 [−2 −33 −8] p < 0.001, see SI Appendix, Supplementary Table 2 for whole-brain results).

In sum, the results of both studies point consistently and specifically to the pgACC as key region during the switch from freezing to shooting under high threat. The amygdala and the PAG were also implicated during shooting, irrespective of threat magnitude however.

### Shooting decisions involve functional connectivity changes along the PAG-amygdala-pgACC axis

Finally, to elucidate how neural circuits involved in preparation interacted with regions associated with the switch to action, we performed effective functional connectivity analyses using psychophysiological interactions (PPI). We predicted increased communication between the anatomically-defined PAG (seed region) on the one hand, signaling preparation for potential shooting under threat of shock, with switch-related regions as the pgACC and the amygdala on the other hand. Regardless of threat magnitude, connectivity between PAG (seed), amygdala and pgACC was increased during preparation (Study 2: amygdala SVC: high threat [24, −6, −14] p = 0.009; low threat [18, 0, −14] p = 0.004, pgACC SVC: high threat [4, 36, 7] p = 0.02, [−22, 33, −5] p = 0.03, [−25, 40, −8] p = 0.03; low threat [1, 36, 10] p = 0.006, see Fig. 3a). During the switch to action, we found increased pgACC(seed)-amygdala connectivity under threat of shock (Study 2: amygdala SVC: high threat [34, 0, −29] p = 0.046, see Fig. 3c; low threat [18, 0, −17] p = 0.09). No other regions were found to be associated with the pgACC seed region, including the PAG. These results were not found in the smaller sample of Study 1, but this study was likely underpowered for this analysis^34^.

Summarizing, shooting decisions involved an upregulation of connectivity within brain regions involved in defensive preparation and those involved in mediating the switch to action.

## Discussion

Primary responders such as police officers encounter threatening situations which necessitate optimal action decisions. By uncovering the neural circuits underlying human shooting decisions under threat of shock, we gained insights into fundamental mechanisms that dynamically control defensive behaviours such as freezing and fight responses. In two independent studies, we demonstrate that shooting decisions and their build-up under threat involve two distinct psychophysiological states. An initial preparatory state of defensive reactions was indexed by postural freezing, bradycardia, midbrain activity including the PAG, and PAG-amygdala connectivity. All these measures are consistent with previous studies on human freezing reactions^7,35,36^. Importantly, this midbrain activation during freezing was linked to enhanced action preparation under high threat by a robust relation with shooting speed, specifically for correct responses. The subsequent switch from preparation to shooting action was associated with tachycardia, pgACC activity and pgACC-amygdala connectivity providing a neural mechanism implicated in switching between key defence modes in humans (see SI Appendix, Supplementary Fig. 7 for schematic overview). These results reveal a core neural circuit for shooting performance under high threat that we show to be active in trained police recruits also. Moreover, these results provide empirical evidence for the role of defensive reactions such as freezing and associated bradycardia in action-dependent decision making.

The finding that freezing and associated bradycardia enhance action preparation is in line with the view that freezing constitutes a decision stage, during which the most optimal defensive action is selected. For example, previous work in rodents indicates that the startle response, a defensive reflex to threat, is potentiated during freezing^37^. By showing that anticipatory midbrain activity, including the PAG, was associated with faster, accurate actions and co-activated with brain regions related to motor preparation (SMA and striatum) under high threat conditions, our data advance the literature and provide a neural mechanism through which threat-anticipatory reactions facilitate the preparation and the execution of a subsequent action.

In line with previous work on human freezing and threat anticipation, we observed heart rate decelerations during threat of shock^16,38^ (but see^39^), and body sway reductions at the same time points of the same task conducted outside the scanner on a stabilometric force platform. Heart rate and body sway also decelerated -to a lesser extend– in low threat trials, where there was no physical threat (of shock) but still threat of visual feedback of being shot by the opponent. Moreover, previous studies also demonstrated phasic bradycardia in anticipation of decisions requiring selective attention without threat^11,40^. Thus, bradycardia seems linked to a state of vigilant anticipation and action preparation characteristic of, but not exclusive to, threat encounters. These findings converge to highlight how freezing-related bradycardia may support defensive decision making, through its’ link not only with increased perception^41^, but also with action preparation. In non-human animals, stimulation studies on autonomic branches support that rapid heart rate changes (on a beat-to-beat interval), similar to what we observed here, are mainly driven by parasympathetic branches, as heart rate modulations from sympathetic branches are considerably slower^42^. Consistent with this, a recent human neuroimaging study demonstrated that PAG activity was associated with freezing-related bradycardia but not with sympathetic pupil dilation^16^. In the current study, parasympathetic engagement at the level of the heart was supported by additional analyses indicating higher vagal tone -as indexed by the Root Mean Square of the Successive Difference- during preparation under high threat that was robustly related to stronger heart rate decelerations (see SI Appendix). Nevertheless, contributions of the sympathetic branch due to baroreceptor-mediated reflexes cannot be ruled out^43^.

This first study in humans on the neural switch from defensive freezing-related reactions to actions converges with findings from work in rodents showing that the CE, together with the mPFC, shift responses to a threatening stimulus from freezing to overt defensive actions^24,26^. Anatomical connections between mPFC regions including pgACC, and subcortical regions like the amygdala and PAG have often been demonstrated in rodents, primates, and humans^44^, making such functional interactions viable. The specificity of the pgACC activation during shooting under high threat was striking, as it was solely and consistently activated within both studies. Functionally, the ACC has been segregated along the ventral/dorsal axis, in which these sub-regions are ascribed to have control of parasympathetic and sympathetic states, respectively^45^. The pgACC region we found, lies exactly between this dorsal/ventral dissociation and may therefore be in an ideal anatomical position to enable fast switching between different autonomic states, such as the switch from parasympathetically-dominated bradycardia to sympathetically-dominated tachycardia. In line with such autonomic accounts, the pgACC has been proposed to play a critical role in salience evaluation and the determination of the magnitude of subsequent responses. This evidence fits well with the threat-related pgACC results we report here. Our findings extend the neuroimaging literature on the role of the pgACC and highlights that humans use similar neural networks as rodents when critical switching between innate defensive behaviours is required.

Those few neuroimaging studies that exist on defensive behaviours in humans mainly focussed on neural mechanisms associated with the (spatio-temporal) imminence of threat^39,46,47^. Our modelling of “fight” actions is different from avoiding capture by “flight” as studied in threat imminence paradigms^47^ but the results converge in demonstrating a role for the putative PAG. Particularly, a recent study showed evidence for the involvement of the PAG during passive (under approaching threat) as well as active avoidance (with more imminent threat) in humans^39^ replicating previous animal work^4,33^. Our study extends these previous findings by considering shifts from the qualitatively different behavioural and autonomic modes of (overtly) passive (freezing) to active (fight-flight) defense^3,4^. To capture this qualitative difference, we show the full cascade from the parasympathetically-driven bradycardia response during freezing that switches to the sympathetically-driven tachycardia response during action.

Our rare set-up with two independent fMRI samples allows for direct testing of the robustness of the observed effects, which is an important asset given recent concerns regarding scientific reproducibility^28^. The behavioural and psychophysiological effects were highly similar in both studies (see SI Appendix), as were all our key findings. Only our findings regarding neural connectivity and the link between heart rate and PAG activity were not reproduced in the smaller sample. While this could theoretically be due to a difference between police officers and the unselected participants, we believe this is an issue of statistical power.

Given the limited spatial resolution of our whole brain BOLD-fMRI measurements, we were not able to dissociate whether the observed PAG activation during preparation originated in the ventral PAG, previously associated with reactive freezing, as opposed to more dorsal parts, found to be related to fight-or-flight responses^4,48^ (for a review see^5^). While the resolution of our whole brain functional imaging makes it also challenging to separate the PAG from surrounding regions, our activation location is highly consistent with anatomical templates of the PAG, as well as with peak coordinates of a meta-analysis focusing on neuroimaging results of the human PAG^32^. Closer inspection (see Supplementary Information) also revealed that our unsmoothed activations are more consistent with a location in the PAG than adjacent nuclei involved in threat processing such as the ventral tegmental area (VTA), raphe nuclei and the superior colliculus (SC)^16,32^. Nevertheless, given the interindividual variety in anatomy and limited resolution of our fMRI scans, contributions of the surrounding nuclei to the activation patterns observed here cannot be fully ruled out. Therefore, high-resolution follow-up studies would be informative for addressing questions concerning the specificity of activity to PAG subregions as well as the role of the directly surrounding areas.

Research on police work has shown that shooting performance drastically decreases from high performance during training to low performance during real-life threatening situations^1^. This calls for an increased understanding of how psychophysiological responses to acute threat drive decision-making as well as performance. Our findings may have at least three implications related to this. First, by showing distinct neural circuits underlying the two discernible critical stages of shooting decisions under high threat, our results provide starting points for training programs aiming to use heart rate and neurofeedback to improve decision-making under threat. Second, they generate testable hypotheses on the brain circuits involved in overly impulsive, aggressive or fearful behaviours in response to threat or prejudice biases^49^ which may involve altered preparatory freezing activity in the PAG as well as altered switch-related activity in the pgACC. Finally, previous studies on shooting performance in soldiers, police officers and athletes in real-life simulations^50^ converge with our psychophysiological and behavioural results, which suggests ecological validity of our experimental task and set-up. Our fMRI results extend this literature by providing neural mechanisms of shooting decisions and the facilitated action preparation during anticipatory freezing, with relatively high anatomical precision of measuring the neural circuitry important for threat.

In conclusion, our results suggest that shooting decisions in the face of threat involve an initial preparatory state of freezing driven by PAG activity, which enhances action preparation. As soon as critical action needs to be initiated, a network including the pgACC and amygdala is involved in the switch to defensive shooting. The interconnectivity of these key neural regions during the two distinct states of shooting decisions suggests a coordinated involvement of this network in exerting fast, accurate actions under acute threat.

## Materials and Methods

All research activities were approved by a local ethics committee (Study 1 by the Ethical Reviewing Board (METC/CMO region Arnhem-Nijmegen, CMO 2013/553), Study 2 by the Independent Review Board Nijmegen (NL48861.072.14)) and were conducted in accordance with these guidelines and regulations. On the same experimental day, before fMRI data acquisition, each participant gave written informed consent and performed the task outside the scanner in a standing poistion on a stabilometric force platform to assess postural freezing (see SI Appendix for Data of Study 2). For figures that include photographs that allow the identification of an individual person, informed consent has been obtained to publish the photograph in an online open-access publication.

### Participants

Participants (aged between 18 and 41 years), reported no history or current condition nor treatment of psychological, neurological or endocrinological disorders. In Study 1, 22 out of 25 nonselected civilians (pre-study) and 54 out of 60 police recruits in training from the Dutch police academy in Study 2 were included in the data analyses (see SI Appendix for details).

### Task

The shooting task (see Fig. 1a) is a speeded decision making task under threat of shock^29^. Trials began with one of two randomly presented opponents (the cue) signaling the preparation for potential action. One of the opponents was associated with threat of an aversive electrical shock (high threat cue) whereas the other signaled shock safety as it was never associated with an electric stimulation (low threat cue). Which opponent was associated to the threat of shock was counterbalanced across participants. After a 500–6500 ms preparation period, the opponent either drew a gun (indexing a shooting response) or a mobile phone (indexing a withhold response). Armed opponents fired the gun after a brief delay forming a response window (500 ms) in which participants could shoot the opponent by a button press before being fired upon. To keep the frequency of aversive stimulations consistent during the task and between participants, the participant’s response window (time between the opponent drawing the gun and firing) was titrated in a way that participants would be shot on +/− 50% of the trials. This was accomplished by an algorithm that dynamically adjusted the duration of the response window (based on the participant’s median reaction time (RT) across low and high threat conditions) resulting in faster or slower firing actions of the opponent. Participants firing too slow were shot by the opponent, whereas participants shooting too early (before the draw) or mistakenly (on withhold trials; false alarms) were shot by a policeman standing in the backside of the garage who was covering the opponent. Only for the high threat cue, these erroneous events were followed with the aversive electrical stimulation. The task involved 75 trials for Study 1 and 70 trials for Study 2 (per high and low threat condition – total 150/140). Note that trials with errors were excluded from the analyses including those that were titrated resulting in about 18 trials for shooting trials for Study 1 and 17 trials for Study 2 per high and low threat condition. For withhold trials, analyses included about 37 trials in Study 1 and 35 trials for Study 2 per high and low threat condition. Before the start of the task, electrical stimulation was set to an unpleasant but not painful level with a standardized work-up procedure (for more details^51^).

### Data analyses

(See SI Appendix for Data Acquisition Details) Event-related heart rate responses (finger pulse photoplethysmograph) were calculated as changes in beats-per-minutes (BPM) for each trial based on inter-beat-intervals (IBIs) relative to a baseline period of 1 second before the event of interest. As distinct autonomic effects were predicted for threat-potentiated anticipatory freezing and shooting actions, we seperated the analyses into two time windows. During the preparation period, we expected freezing-related bradycardia, the analysis was therefore locked to the cue. Action-related tachycardia was predicted after the onset of the draw signaling the need to shoot or withhold, analysis was therefore time-locked to the onset of the draw including a zero-centered baseline correction to analyse BPM changes in the presence of preceding bradycardia. Repeated-measure ANOVA with cue (high vs. low threat) and time was run as within-subject factors seperately for the preparation and draw period. Shooting actions (as opposed to withhold responses) under high threat were predicted to elicit stronger tachycardia as well, we therefore ran repeated measures ANOVA including draw (shoot vs. withhold), cue (high vs. low threat) and time as within-subject factors.

Behavioural analyses aimed to test for threat-potentiated changes on shooting performance including accuracy and reaction times (RT). Paired sample t-statistics tested for differential effects of high vs. low threat trials on RT and accuracy. Additional, repeated-measures ANOVA tested for cue (high vs. low threat) and draw (shoot vs. withhold) interactions on accuracy.

For the fMRI analyses, one of the aims was to identify brain circuits associated with anticipatory freezing reactions and the switch to action amplified by threat magnitude. Based on our hypothesis on the role of the PAG in freezing-related action preparation, we built an anatomical mask based on a meta-analysis of human imaging findings of the PAG^32^. A similar hypothesis on the role of the amygdala during the switch to action led to the use of a prespecified bilateral amygdala mask based on Anatomical Automatic Labeling (AAL). In line with a recent study in rodents also showing the involvement of local PAG circuits in mediating the selection of overt active and passive defensive reactions^33^, we also included a region-of-interest analysis of the PAG during the switch to action. Additionally, effective connectivity changes were predicted between these regions-of-interests during preparation and action. To signal action preparation, the PAG was expected to communicate to the amygdala and mPFC (during preparation). To execute a switch to action, the mPFC was hypothesized to show increased connectivity with the amygdala and the PAG (during shooting). As we had specific apriori hypotheses on these regions, small volume corrections were performed for the target regions in these analyses and were reported after surviving peak thresholds of p < 0.05 FWE corrected for multiple-comparisons (find step-by-step details on all analyses in SI Appendix).

### Relation freezing and action preparation

To investigate whether threat-anticipatory PAG activity during freezing was associated with action preparation, we ran a correlational analysis between PAG activity during preparation and subsequent performance including reaction time and accuracy. As additional indication that this PAG activity was related to freezing, we tested for the relationship between movement cessation, as assessed with body sway reductions and action preparation outside the scanner during the same task (see SI Appendix). To align with previous neuroimaging studies on human freezing, we also tested for the relationship between the PAG and bradycardia inside the scanner. We employed similar analysis to explore whether threat-potentiated switches to shooting actions in the pgACC related to tachycardia. For this purpose, heart rate responses were averaged over the whole analysis time window for the preparation period and the switch period seperately. All correlational analysis was performed using a nonparametric test of Spearman’s rho with alpha set 0.05.

### Power analyses

For analyses that did not reach significance in the smaller sample (Study 1), but did reach formal significance in the larger Study 2 we performed a post-hoc power analysis for Study 1 using the effect size estimation taken from Study 2 (with the power analyses program Gpower power^52^).

### Code Availability

The code that has resulted in the reported findings is available from the corresponding author upon reasonable request.

## Supplementary information

Supplementary Information

## Data Availability

The datasets analysed during the current study are available from the corresponding author upon reasonable request.
