# Supplementary material for: Neural Dynamics of Shooting Decisions and the Switch from Freeze to Fight
Source: Sci Rep. 2019 Mar 12;9:4240. doi: 10.1038/s41598-019-40917-8 (PMC6414631; doi:10.1038/s41598-019-40917-8)
Supplement: Supplementary file 1 — Supplementary Information [file 41598_2019_40917_MOESM1_ESM.pdf]

Supplementary information

Neural Dynamics of Shooting Decisions and the Switch from Freeze to Fight

Mahur M. Hashemi[1,2], Thomas E. Gladwin[1,3], Naomi M. de Valk[1], Wei Zhang[1,2], Reinoud Kaldewaij[1,2], Vanessa van Ast[1,4], Saskia B.J. Koch[1,2], Floris Klumpers[1,2#], Karin Roelofs[1,2#]

Methods

Both experiments were conducted at the Donders Institute for Neuroimaging in Nijmegen, the Netherlands. Participation was financially compensated.

Participants

All subjects were asked to abstain from the use of medication and alcohol 24 hours preceding the experimental session. Technical problems, data loss or discontinuation of task due to sleepiness led to exclusion of participants, which resulted in the inclusion of 22 out of 25 nonselected civilians in Study 1 (8 males and 14 females, pre-study) and 54 out of 60 police recruits in training from the Dutch police academy in Study 2 (43 males and 11 females). The sample size of the initial Study 1 was based on the assumption that around 10% of participants were expected to drop out. The most common approach to estimate the number of participants for sufficient statistical power in fMRI studies is based on previous studies with related topics[1,2]. Additionally, we performed numerical simulations based on different noise assumptions, which also indicated a total number of 20 to 30 subjects in order to have reasonable power (around 80%, at a false-positive rate of 5%). The sample size of Study 2 was based on the effects observed in sample 1. By including a sample size that was at least double the number of the initial study, we were confident we had sufficient power to reproduce any real effects found in the initial study as well as to perform the functional connectivity analyses.

Task

Stimuli. Participants were presented with a parking garage including an opponent avatar at the centre of the screen, an armed policeman in the background (per block alternatingly on the left or right of the screen), and a view of the participant's own "in-task" hands, holding a gun. Two visually distinctive opponents were used.

Task parameters. Before fMRI measurments, a behavioural version of the shooting task, including 350 trials, occurred outside the scanner in a standing poistion on a stabilometric force platform (data of Study 2 reported in Supporting Information). The final measurement phase in the MRI scanner consisted of 180 trials in Study 1 and 140 trials

in Study 2. To acquire a sufficient number of trials for which the time course of preparation-related freezing responses could be analysed, 80% of the preparation period consisted of long trials (Study 1: 5-7 secs, Study 2: 6-6.5 secs). Short (0.5-1.5s) and middle preparation intervals (1.5-6s) were still presented in the rest of the trials, so that the moment of attack was unpredictable, and that activity upon cue and stimulus could be dissociated with fMRI. The inter-trial intervals were set to vary in between 5 to 8 seconds.

Heart rate recording and analyses

Heart rate was recorded during scanning using a finger pulse photoplethysmograph attached to the left index finger. Matlab2015a and SPSS19 were used for data preprocessing and analysis.

Raw pulse data were downsampled to 250Hz and filtered with a butterworth band-pass filter (0.5-10 Hz). Heart rate was assessed via an in-house developed automatized peak detection algorithm. Peak detection was visually inspected trial-by-trial and corrected manually whenever required. Only trials with sufficient duration of minimally 6 seconds were included in the analysis. As electrical stimulation and wrong responses coincided within the switch period, only trials with correct responses and without electrical stimulation were included in the analysis. The first trial from each block, and trials with insufficient signal-to-noise ratio were discarded from the analysis. A minimum of 12 remaining artefact free trials for each condition were necessary to include a participant's dataset within the analysis. Based on this criterion, 18.2% of Study 1 and 24.1% of Study 2 datasets had to be excluded for the preparation period; 40.9% of Study 1 and 25.9% of Study 2 had to be discarded for the switch period. The high exclusion rate for the switch period is likely a result of movement-related artefacts on our finger-clip recordings that were induced by button presses and electrical stimulations to the fingers that coincided in the same time period.

Changes in cardiac responses were calculated in beats-per-minutes (BPM) relative to a baseline period of 1 second before the event onset. We seperated the analyses into two time windows, as we expected different autonomic effects during preparation and action. First, during the preparation period we expected freezing-related

bradycardia, we therefore time-locked this analysis to the cue. To exclude orienting effects on freezing measures[3], changes in BPM were calculated between 3 to 6 seconds relative to the baseline period before cue onset. Second, action-related tachycardia was predicted after the onset of the draw signaling the need to shoot or withhold. For this, we time-locked heart rate analysis to the onset of the draw extending to 3 seconds because the time resolution of sympathetic effects on heart rate were previously described as rather slow (in seconds). To analyse heart rate change in the presence of preceding bradycardia, we performed a baseline correction (including zero-centering) at the time of the draw. Before statistical analysis, time windows were seperated into half-second bins for each condition[4].

To assess whether preparation under high threat (threat of shock) led to stronger bradycardia, during human freezing reactions, we ran a repeated-measure ANOVA with cue (high vs. low threat) and time (split over 7 time windows centered around the following time points: 3.0s, 3.5s, 4.0s, 4.5s, 5.0s, 5.5s, 6.0s post cue) as within-subject factors. Shooting actions (as opposed to withhold responses) under high threat were predicted to elicit stronger tachycardia. We therefore ran repeated measures ANOVA including draw (shoot vs. withhold), cue (high vs. low threat) and time (split over 6 time windows centered around the following time points: 0.5s, 1.0s, 1.5s, 2.0s, 2.5s, 3.0s post draw) as within-subject factors. As we were specifically interested whether shooting under high threat resulted in stronger tachycardia responses, a similar approach was employed for shoot trials only with a repeated measures ANOVA including cue (high vs. low threat)and time (split over 6 time points: 0.5s, 1.0s, 1.5s, 2.0s, 2.5s, 3.0s post draw) as within-subject factors. When assumptions of sphericity were violated, degrees of freedom were corrected using Greenhouse-Geisser estimates. Planned paired sample t-tests were performed to quantify main effects of threat magnitude per time point. Additional nonparametric Wilcoxon signed-rank tests were performed where heart rate data were non-gaussian in order to minimize any potential non-normality and outlier concerns. Main effects of time, threat and action are omitted for brevity, given our specifc interest in the threat-by-time interactions**.**

Behavioural analysis

Behavioural analysis was performed with Matlab2015a and SPSS19. We predicted threat-induced changes on shooting performance including accuracy and reaction times (RT). Trials in which participants were shot due to the titration were discarded from the analysis. RT analyses involved only correct responses within the response window of 200-500ms. Paired sample t-statistics tested for differential effects of high and low threat trials on RT. A repeated-measures ANOVA tested for cue (high vs. low threat)and draw (shoot vs. withhold) interactions on accuracy. The interaction effects were our main interest, hence only those were reported here. Planned paired-sample t-tests were used to compare the effect of threat of shock against shock safety. Again, nonparametric Wilcoxon signed-rank tests were used where behavioural measures were not normally distributed in order to reduce any potential non-normality and outlier concerns.

fMRI acquisition

Whole brain T2*-weighted BOLD fMRI data was acquired using echoplanar imaging (EPI) on a Siemens 3T system (Study 1: Magnetom skyra system, Study 2: Magnetom Prisma Fit system) using an ascending slice acquisition sequence (Both studies: 37 slices, 3mm slice thickness, matrix size = 64*64, field of view (FOV) = 212 x 212 x 122 mm, flip angle = 90°, voxel size = 3.3 x 3.3 x 3.0 mm, echo time (TE)1 = 11.0 ms, TE2 = 25 ms (multiecho), repetition time (TR) for Study 1: 1730 ms, for Study 2 :1740ms). The choice of a multiecho sequence was based on its increased sensitivity to BOLD and decreased susceptibility to artefacts in ventral brain regions such as the PAG and amygdala[5]. The first volumes (Study 1: 31, Study 2: 5) were discarded to allow for T1 equilibration. High-resolution structural images in both studies were obtained using a Magnetization Prepared Rapid Gradient Echo (MPRAGE) scan combined with GeneRalized Autocalibrating Partially Parallel Acquisitions (GRAPPA) (192slices, FOV = 256 x 256 x 192 mm, voxel size = 1.0 x 1.0 x 1.0 mm, TR = 2300 ms, TE = 3.03 ms, inversion time (TI) = 1100 ms, flip angle = 8 degrees, GRAPPA acceleration factor = 2).

fMRI preprocessing

Image Preprocessing and analysis were performed using Matlab2012b and SPM8 (http://www.fil.ion.ucl.ac.uk/spm; Welcome Department of Imaging Neuroscience,

London, UK). Volume-to-volume realignment parameters were calculated from the shortest TE-images and applied to all other TEs[5] using a least squares approach and a six parameter (rigid body) spatial transformation[6]. Thirty volumes were used to estimate weighting images representing BOLD contrast-to-noise ratio maps for each TE. Based on those images and their weighted summation, the two echos were combined to give an optimal signal intensity for each individual voxel, especially in the regions around the cavities where normally signal drop-out is observed[5]. Functional images were then coregistered to the bias-corrected structural image. Structural images were segmented and normalized to the Montreal Neurological Institute 152 T1-template image using the gray matter for calculation of the nonlinear transformation matrix. This matrix was used to normalize all functional images which were then resampled into 3.3 x 3.3 x 3 mm voxels. Spatial smoothing was performed with an isotropic Gaussian kernel of 6 mm full-width at half maximum.

fMRI analyses

One of our main aims was to identify brain circuits associated with freezing responses (preparation period) and the switch to action under acute high threat. First level event-related analyses were conducted in the context of a general linear model (GLM). We built independent regressors modeling the cue (high and low threat cues) and the draw (shooting and withhold responses) as main task regressors. Additional regressors as erroneous responses (shooting too soon, too late or falsely), electric stimulation and button presses were modeled seperately to maximally explain variance. The preparation period was modeled as boxcar function whereas remaining short-term events were modeled as stick functions. Regressors were then temporally convolved with the canonical hemodynamic response function (HRF) of SPM8. Six movement parameters (3 translations, 3 rotations) as well as a high-pass filter with a cut-off of 128 seconds were added as nuisance regressors. In Study 2, inspection of the fMRI signal reflected sudden slice specific fluctuations in signal intensity that were too fast to be blood oxygen level dependent (BOLD). To ensure that these signal intensity differences did not bias results, we included regressors modeling slice-specific global signal intensities (one per slice). In Study 1, we did not observe any slice-specific fluctuations and therefore included only 2

(global brain and out-of-brain) signal intensity regressors to model additional movement-related nuisance.

Contrast images were generated at the single-subject level, focussing on threat of shock vs. shock safety during the preparation, and the switch to action. These main task contrasts were then entered into a group-level random effects (RFX) analysis using one-sample-t-tests. Statistical parametric maps were initially thresholded to p<0.005 (uncorrected voxelwise) and BOLD activations at the whole brain level were reported significant that survived p<0.05 Family-Wise Error (FWE) cluster-corrected for multiple comparisons. As we had a apriori hypothesis on the role of the PAG in freezing-related action preparation, we built an anatomical mask based on a meta-analysis of human imaging findings of the PAG[7]. A box around the peak-coordinates [1, -29, -12] was built with a width of x = 10, y = 7 and z = 10mm. We chose a slightly larger window so that anatomically-tilted voxels of the PAG would still be included within the mask. During the switch to action, a similar hypothesis on the role of the amygdala led to the use of a prespecified bilateral amygdala mask based on Anatomical Automatic Labeling (AAL). In line with a recent study in rodents also showing the involvement of local PAG circuits in mediating the selection of overt active and passive defensive reactions[8], we also included a region-of-interest analysis of the PAG during the switch to action. Within these regions of interest, significant BOLD activations were only reported after surviving peak thresholds of p<0.05 FWE small-volume corrected[9].

Functional connectivity analyses

To explore how neural circuits involved in defensive preparation interacted with regions recruited during the switch to action under high threat, we implemented two psychophysiological interactions analyses (PPIs). Specifically, we investigated event-related connectivity first during preparation using PAG as seed region (contrasting preparation under high vs. low threat, preparation under high threat vs. baseline, preparation under low threat vs. baseline), and second during the switch to action with the pgACC as seed region (contrasting switch upon shooting under high vs. low threat, switch to shooting under high threat vs. baseline, switch to shooting under low threat vs.

baseline). For the seed region in the pgACC, we built a functional ROI based on Study 1's activation cluster found during threat-potentiated switches to actions.

At single-subject level, the HRF was first deconvolved and then the signal was extracted from the seed region. The extracted signal timecourses were then integrated in the GLM as main effects regressor together with an interaction term (seed region modulated by experimental context). Similar nuisance regressors as mentioned in the first analysis were also added to the GLM. The interaction-terms were then entered into a group-level random effects (RFX) analysis in the form of a one sample t-test.

Based on apriori hypothesis and activation results, we predicted that during the preparation period, the PAG communicates to the amygdala and the pgACC to signal action preparation. To execute a switch to action, the pgACC is hypothesized to show increased connectivity with the amygdala and the PAG (during shooting). As we had specific apriori hypotheses on these regions, small volume corrections were performed for the target regions in these analyses and significant functional connectivity was reported after surviving peak thresholds of $p<0.05$ FWE corrected for multiple-comparisons.

Results

To verify that the results of enhanced heart rate acceleration in the high threat condition would not merely be a result of the enhanced deceleration we observed during preparation, we repeated this analysis and used a baseline correction before the cue rather than at the draw. This control analysis again returned a significant cue-by-time interaction on shooting trials (cue [high vs. low threat] by time [relative to cue onset] Study 1 $F_{(2.84, 34.05)} = 4.54$ $p=0.01$; Study 2 $F_{(2.37, 92.37)} = 8.31$ $p=0.000$). This shows that the heart rate accelerated beyond the level of the low threat stimulus, thus ruling out that the acceleration was a mere stronger "returning to baseline" in the high threat condition.

Supplementary Figure S1. Shooting decisions under high threat amplified bradycardia during preparation, and tachycardia during the switch to action.

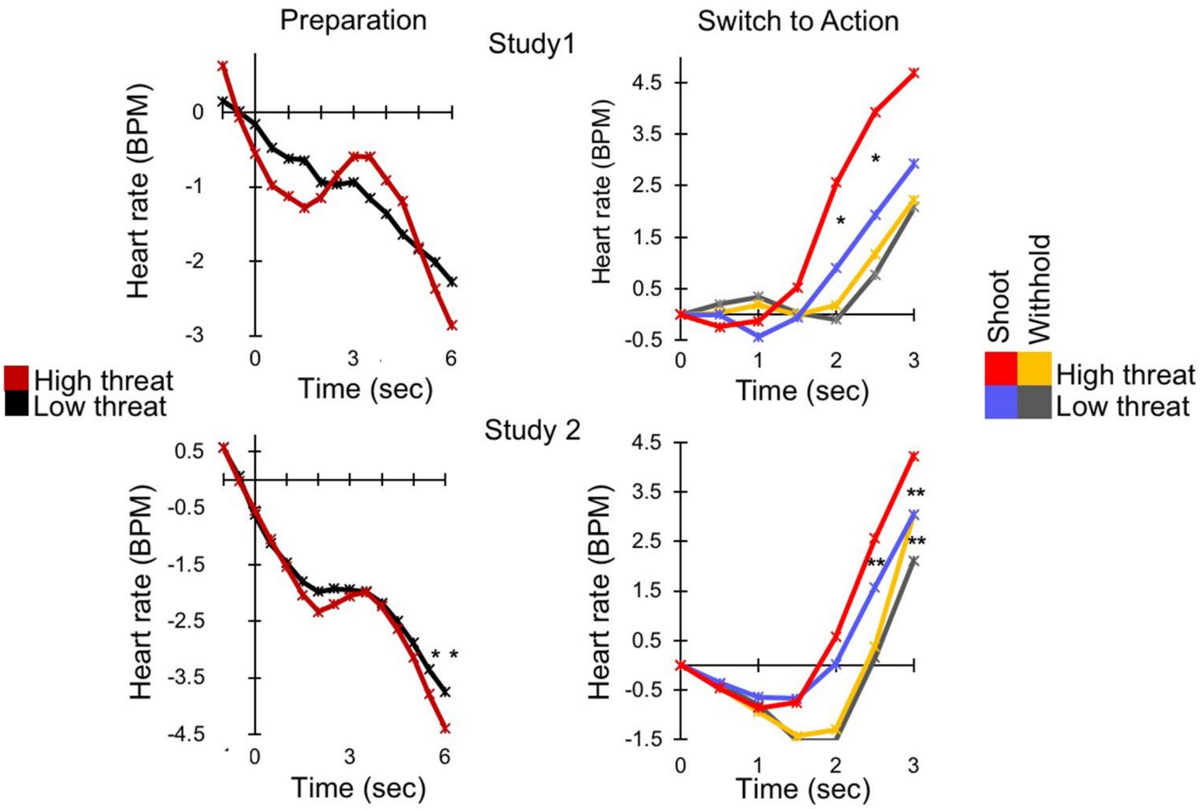

Average heart rate in beats-per-minute (BPM) across participants for Study 1 (top) and Study 2 (bottom) shows heart rate deceleration during preparation (Study 1: N=18, Study 2: N=42) (left) and heart rate acceleration during the switch to action (Study 1: N=13, Study 2: N=40) (right). Heart rate patterns were amplified under threat of shock (compared to safety) during preparation as well as during the switch to shooting action. Shooting compared to withhold responses also accelerated heart rate responses. Asterisks indicate pair-wise significance of high vs. low threat trials (Paired sample t-test for Study 1, and Wilcoxon signed-rank test for Study 2) * = $p<0.05$, ** = $p<0.01$, *** = $p<0.001$.

Stronger movement cessation during preparation under high threat

To verify freezing reactions, we recorded displacements of the centre of pressure on a force platform during the same task outside the scanner in Study 2. In line with previous research (see methodological details[3,10,11]), we calculated the standard deviation of the centre of pressure in the anterior-posterior direction –as the index of bodily freezing-

during the preparation period. Similar to our heart rate findings, we observed reductions in body sway during both high and low threat conditions (time main effect [locked to the onset of the cue] Study 2: $F_{(1.6,\ 84.74)}$ p=0.000; cue [high vs. low threat] x time [locked to the onset of the cue] Study 2: $F_{(4,17,\ 221.18)}$ = 1.84 p=0.12). Crucially, we found a significantly stronger reduction in body sway during preparation under high threat (time point 5.5s; Wilcoxon signed-rank test p=0.01, other time points p>0.05, see Supplementary Fig. S2). Lastly, body sway reductions correlated with heart rate decelerations under high threat within this behavioural task session across participants (Rs=0.37, p=0.006).

Supplementary Figure S2. Stronger movement cessation during preparation under high threat

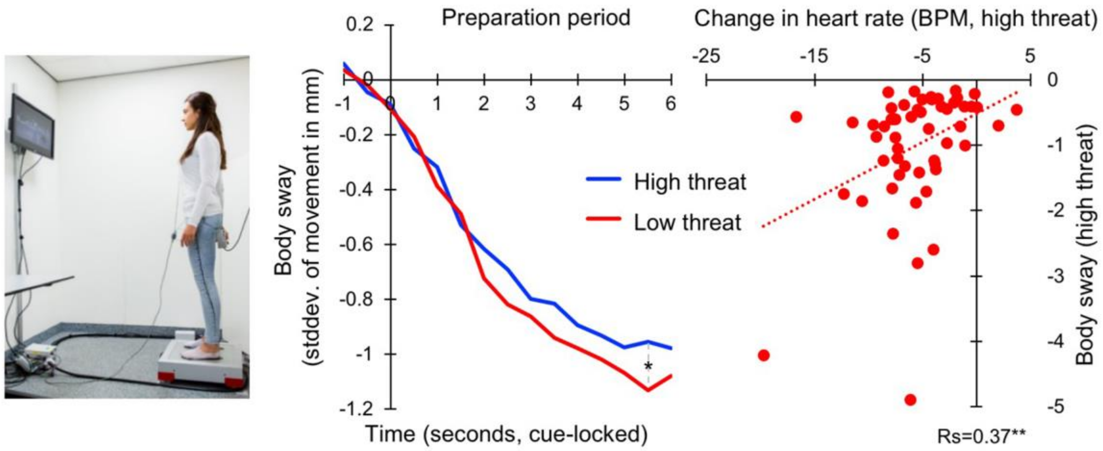

Mean body sway across participants during preparation is reduced under high threat compared to low threat trials (left). Body sway is expressed in standard deviation (stddev.) of movement from the centre of pressure in the anterior-posterior direction. The asterisk indicates pair-wise significance for high vs. low threat trials (Wilcoxon signed-rank test for Study 2, * = p<0.05). Stronger body sway reduction is associated with stronger heart rate deceleration during preparation under high threat (right). Rs = two-sided Spearmans Rho, ** = p<0.01.

Supplementary Figure S3. Trigger-prone behaviour during high threat in Study 1

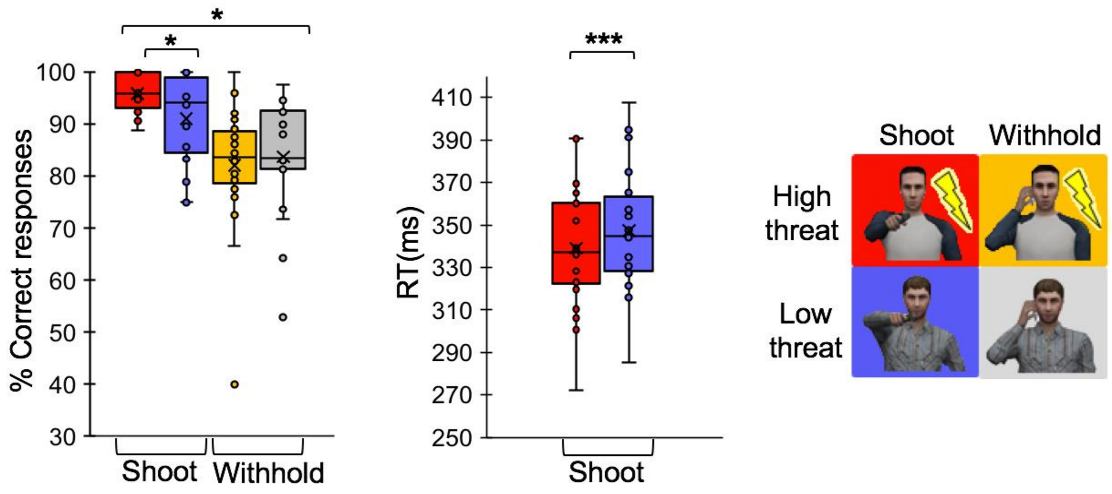

Threat of shock elicited faster and more accurate reactions when shooting was required. Study 1 (N=22) is illustrated here, and highly similar results for Study 2 can be found in Fig. 2 of the main text. Additional non-parametric Wilcoxon signed-rank tests were performed to minimize any potential non-normality and outlier concerns. Asterisks indicate pair-wise significance (Paired sample t-test for RT, and Wilcoxon signed-rank test for accuracy) * = $p<0.05$, *** = $p<0.001$.

Supplementary Table 1: Peak voxels and related t-values of activation clusters during preparation of shooting (threat of shock > shock safety).

	Study1					***Study2***				
Region	***Cluster size***	***x***	***y***	***z***	***t***	***Cluster size***	***x***	***y***	***z***	***T***
Midbrain*	572	11	-10	4	6.08	612	11	10	7	5.41
SMA extending to dACC	154	8	-3	58	4.92	1493	4	-6	58	7.43
Supra marginal gyr.	78	61	-46	31	5.41					
Temporal mid gyr.	71	54	-59	10	4.99	395	47	-59	10	4.95
Occipital	114	14	-69	13	5.15	270	-48	-69	4	5.29
	87	-15	-86	4	4.70					

All coordinates are defined in MNI space and brain results are $p<0.05$ FWE cluster-corrected for multiple comparisons. * extending to thalamus, hypothalamus, PAG and striatum (including BNST).

Supplementary Table 2: Peak voxels and related t-values of activation clusters during shooting (shooting > withholding).

	Study1					*Study2*				
Region	***Cluster size***	***x***	***y***	***z***	***t***	***Cluster size***	***x***	***y***	***z***	***T***
dACC extending to central gyr.	5238	-5	-16	52	8.05	8400	-	-20	55	8.76
		41	-3	31			38	-13	43	
		28	-30	58			37	-16	43	
							-9			
Occipital	79	-22	-79	25	5.49	146	24	-76	1	5.00
							28	-72	34	
							31	-72	19	
Medial PFC	85	-9	43	-14	3.98					
		11	36	-14						
		1	43	-14						

All coordinates are defined in MNI space and brain results are p<0.05 FWE cluster-corrected for multiple comparisons.

Supplementary Figure S4. Whole brain activation overlap of both studies during preparation under threat of shock compared to shock safety trials.

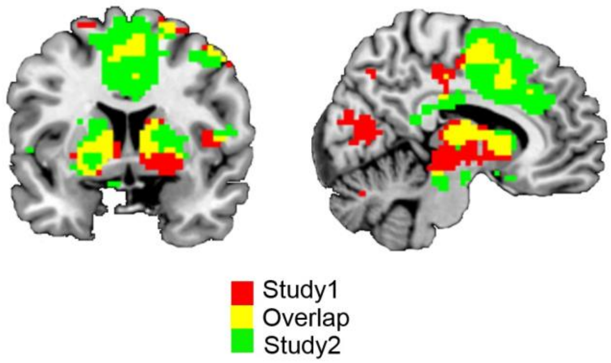

Activation overlap (yellow) shown for Study 1 (N=22, red) and Study 2 (N=54, green). Preparation under threat of shock (contrasted to shock safety trials) activated brain regions associated with threat appraisal and action preparation including the midbrain,

striatum, supplementary motor cortex, dorsal anterior cingulate cortex. All these brain results reported are p<0.05 FWE whole brain corrected for multiple comparisons. For a frank view of the overlap in activity, results are illustrated here with an uncorrected threshold of p<0.01.

Supplementary Figure S5. Stronger PAG activity during preparation under threat of shock is linked to bradycardia.

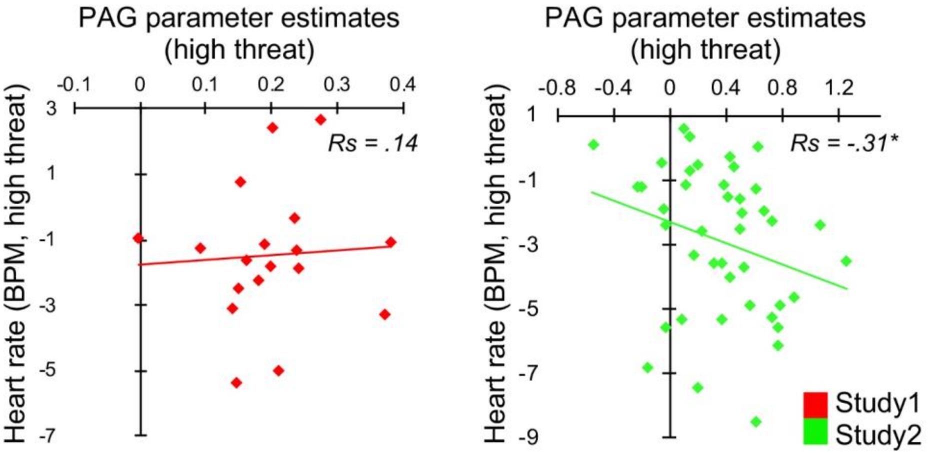

In Study 2 (N=42, right, green), increased PAG activity during preparation under threat of shock correlated with stronger heart rate decelerations. A similar relationship was not found in Study 1 (N=18, left, red) although this might be explained by lack of power in this smaller sample. Rs= two-sided Spearmans Rho, asterisk indicates statistical significance of p<0.05.

Source of peak activation in midbrain

To investigate the source of peak activations relative to neighbouring structures more in depth, we additionally tested for peak voxels within a bilateral anatomical mask for the SC (3mm sphere with coordinates centring around [0,-30,-4][12], see also[13]). We did this for the SC especially because of its anatomical proximity to our activation. This analysis suggests part of our activation spread to the SC (next to the PAG) in Study 1, but not in the larger Study 2 where the activation was more ventral.

Beat-to-beat heart rate variability during preparation

The Root Mean Square of the Successive Differences (RMSSD) assesses beat-to-beat variability and is commonly used to measure vagal tone[14]. We therefore added this analysis to show the involvement of the parasympathetic system in heart rate decelerations in Study 2. Although this measure is typically applied on longer recording epochs, previous studies have shown the validity of ultra-short recordings to estimate RMSSD[15]. We therefore estimated the median RMSSD within the preparation period of minimally 6 seconds for each trial, and calculated the mean over all trials for high and low threat conditions separately. We indeed observed increased RMSSD values, and therefore higher vagal tone during preparation under high threat compared to low threat trials (Study 2: mean low threat condition = 47.64; mean high threat condition = 49.46), although perhaps due to the short time-frame only at trend-level (p=0.08, two-tailed) significance. Further support for the contribution of the parasympathetic tone comes from correlational analyses showing that higher RMSSD values are significantly associated with stronger heart rate decelerations across high and low threat conditions with a moderate effect size (Study 2: high threat: Rs = -.59; low threat: Rs = -.56, p<0.001, see Supplementary Fig. S6).

Supplementary Figure S6. Beat-to-beat heart rate variability during preparation

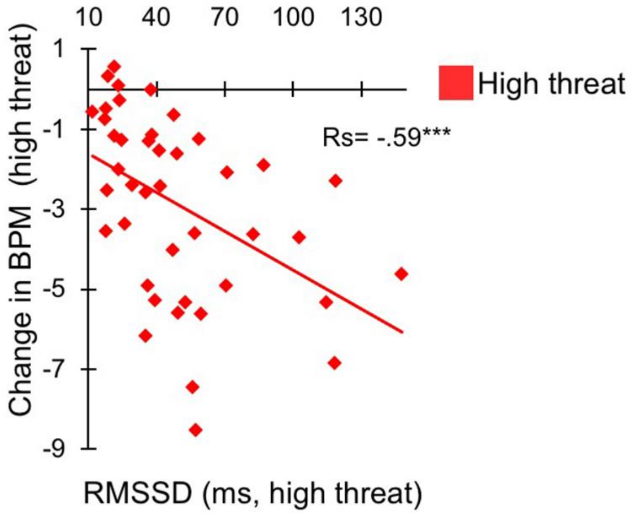

Higher Root Mean Square of the Successive Differences (RMSSD) values were significantly related to stronger heart rate decelerations as estimated in beats-per-minutes

(BPM) on high threat trials (right), a similar link was found on low threat trials. (Rs = Spearmans Rho, *** = p<0.001).

Supplementary Figure S7. Schematic overview of the main findings of this study.

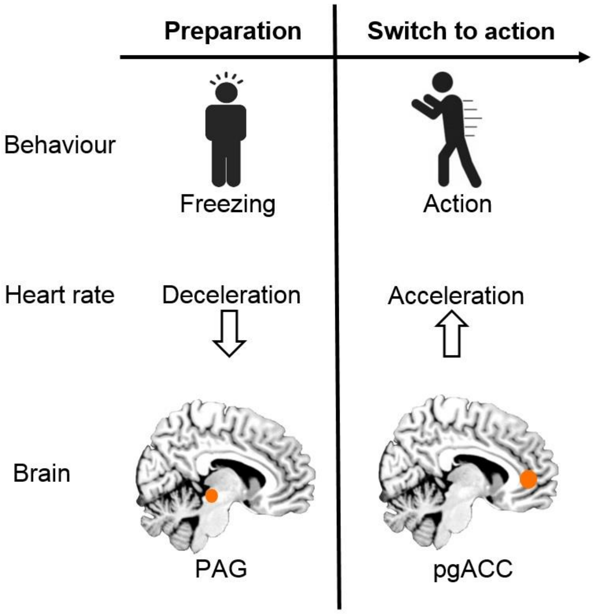

Defensive preparation is associated with bodily freezing, heart rate deceleration and threat-enhanced periaqueductal gray (PAG) activity (left). The switch to action is linked to heart rate acceleration as well as threat-enhanced perigenual anterior cingulate (pgACC) activity (right).

References

1. Hermans, E. J., Henckens, M. J. A. G., Roelofs, K. & Fernández, G. Fear bradycardia and activation of the human periaqueductal grey. *Neuroimage* **66,** 278–287 (2013).
2. Mobbs, D. *et al.* From Threat to Fear: The Neural Organization of Defensive Fear Systems in Humans. *J. Neurosci.* **29,** 12236–12243 (2009).
3. Hagenaars, M. A., Roelofs, K. & Stins, J. F. Human freezing in response to affective films. *Anxiety. Stress. Coping* **27,** 27–37 (2014).
4. Graham, F. K. Constraints on measuring heart rate and period sequentially through real and cardiac time. *Psychophysiology* **15,** 492–495 (1978).
5. Poser, B. A., Versluis, M. J., Hoogduin, J. M. & Norris, D. G. BOLD contrast

sensitivity enhancement and artifact reduction with multiecho EPI: Parallel-acquired inhomogeneity-desensitized fMRI. *Magn. Reson. Med.* **55,** 1227–1235 (2006).

6. Friston, K. J., Penny, W. D. & Glaser, D. E. Conjunction revisited. *Neuroimage* **25,** 661–667 (2005).
7. Linnman, C., Moulton, E. A., Barmettler, G., Becerra, L. & Borsook, D. Neuroimaging of the periaqueductal gray: State of the field. *Neuroimage* **60,** 505–522 (2012).
8. Tovote, P. *et al.* Midbrain circuits for defensive behaviour. *Nature* **534,** 206–212 (2016).
9. Worsley, K. J. *et al.* A unified statistical approach for determining significant signals in images of cerebral activation. *Hum. Brain Mapp.* **4,** 58–73 (1996).
10. Gladwin, T. E., Hashemi, M. M., van Ast, V. & Roelofs, K. Ready and waiting: Freezing as active action preparation under threat. *Neurosci. Lett.* **619,** 182–188 (2016).
11. Roelofs, K., Hagenaars, M. A. & Stins, J. Facing freeze: social threat induces bodily freeze in humans. *Psychol. Sci.* **21,** 1575–1581 (2010).
12. Himmelbach, M., Linzenbold, W. & Ilg, U. J. Dissociation of reach-related and visual signals in the human superior colliculus. *Neuroimage* **82,** 61–67 (2013).
13. Krebs, R. M. *et al.* High-field fMRI reveals brain activation patterns underlying saccade execution in the human superior colliculus. *PLoS One* **5,** (2010).
14. Chambers, A. S. & Allen, J. J. B. Cardiac vagal control, emotion, psychopathology, and health. *Biol. Psychol.* **74,** 113–115 (2007).
15. Munoz, M. L. *et al.* Validity of (Ultra-)Short recordings for heart rate variability measurements. *PLoS One* **10,** 1–15 (2015).
